# Postoperative Capecitabine with Concurrent Intensity-Modulated Radiotherapy or Three-Dimensional Conformal Radiotherapy for Patients with Stage II and III Rectal Cancer

**DOI:** 10.1371/journal.pone.0124601

**Published:** 2015-04-27

**Authors:** Ning-Ning Lu, Jing Jin, Shu-Lian Wang, Wei-Hu Wang, Yong-Wen Song, Yue-Ping Liu, Hua Ren, Hui Fang, Xin-Fan Liu, Zi-Hao Yu, Ye-Xiong Li

**Affiliations:** Department of Radiation Oncology, Cancer Hospital, National Cancer Center, Chinese Academy of Medical Sciences (CAMS) and Peking Union Medical College (PUMC), Beijing 100021, P. R. China; Taipei Medical University, TAIWAN

## Abstract

**Background:**

The aim of this study was to evaluate the survival outcomes and toxicity of postoperative chemoradiotherapy with capecitabine and concurrent intensity-modulated radiotherapy (IMRT) or three-dimensional conformal radiotherapy (3D-CRT) in patients with stage II and III rectal cancer.

**Patients:**

We recruited 184 patients with pathologically proven, stage II or III rectal cancer. Following total mesorectal excision (TME), the patients were treated with capecitabine and concurrent IMRT/3D-CRT. The treatment regimen consisted of two cycles of oral capecitabine (1600 mg/m^2^/day), administered twice daily from day 1–14 of radiotherapy, followed by a 7-day rest. The median pelvic dose was 50 Gy in 25 fractions. Oxaliplatin-based adjuvant chemotherapy was administered after the chemoradiotherapy.

**Results:**

The 5-year overall survival, disease-free survival and locoregional control (LRC) rates were 85.1%, 80% and 95.4%, respectively. Grade 3 and 4 toxicities were observed in 28.3% of patients during treatment. Grade 3 or 4 late toxicity, including neurotoxicity or gastrointestinal toxicity, was only observed in nine patients (4.9%).

**Conclusions:**

This study demonstrated that capecitabine chemotherapy with concurrent IMRT/3D-CRT following TME is safe, is well tolerated and achieves superior LRC and favorable survival rates, with acceptable toxicity.

## Introduction

Rectal cancer is a common malignancy worldwide [1]. Several randomized trials have demonstrated improved locoregional control (LRC) and survival rates with preoperative or postoperative chemoradiotherapy in patients with stage II and III rectal cancer [2–7]. Compared to postoperative chemoradiotherapy, preoperative chemoradiotherapy is associated with lower treatment-related toxicity, less local recurrence and improved disease-free survival (DFS) rates, and may enhance sphincter preservation [8–10]. Although preoperative chemoradiotherapy is considered the standard treatment for patients with locally advanced rectal cancer, postoperative chemoradiotherapy remains the choice of treatment for such patients [10, 11].

Concurrent chemoradiotherapy with 5-fluorouracil (5-FU) is a recommended treatment for stage II and III rectal cancer [2, 3]. However, preliminary trials in patients with metastatic colorectal cancer have indicated that capecitabine may be as effective as standard 5-FU-based chemotherapy in terms of progression-free survival (PFS) and overall survival (OS) rates, while offering a better tolerability profile and a lower incidence of stomatitis [12–18]. Based on these reports, we carried out a phase I trial to evaluate the maximum tolerated dose (MTD) of capecitabine with concurrent postoperative radiotherapy for stage II and III rectal cancer. Consistent with other studies [19, 20], the MTD was 1600 mg/m^2^/day [21].

Advanced radiation techniques, such as three-dimensional conformal radiotherapy (3D-CRT) and intensity-modulated radiotherapy (IMRT), are widely used in the treatment of other cancers. Recent dosimetric studies have demonstrated that IMRT/3D-CRT can provide superior target dose distribution and normal tissue protection in patients with rectal cancer [22–26]. However, the long-term outcome of patients with rectal cancer treated by IMRT/3D-CRT has not been determined; therefore, this study was conducted to investigate the toxicity and long-term survival rates of capecitabine and concurrent IMRT/3D-CRT in patients with stage II and III rectal cancer.

## Patients and Methods

### Patient selection and evaluation

Patients in this study were retrospectively selected from our centre. The eligibility and exclusion criteria were similar as the previous phase I study, except for the requirements of IMRT/3D-CRT [21]. Briefly, patients aged 18–75 years with postoperatively pathological confirmed as stage II or III rectal adenocarcinoma with negative recision margin, normal biochemical examinations and good performance status (ECOG 0–1) were recruited for this retrospective analysis. Patients with a history of chemotherapy or radiotherapy were excluded. Patients with significant comorbidities, uncontrolled infections, pregnancy or second primary tumor *in situ*, other than non-melanoma skin cancer or cervical carcinoma, were also excluded. The study regimen was approved by the Institutional Ethics Committees of Cancer Hospital, Chinese Academy of Medical Sciences, and all patients gave written informed consent prior to the treatment in accordance with the declaration of Helsinki.

Prior to enrollment, a medical history was obtained for each patient, and a physical examination was performed, including blood counts, serum biochemistry, pregnancy testing, chest radiography and a computed tomography (CT) scan of the chest, abdomen and pelvis. A total of 184 patients who had been diagnosed with stage II or III rectal cancer and treated with postoperative concurrent chemoradiotherapy with IMRT/3D-CRT technique between March 2005 and January 2011 were selected and retrospectively analyzed.

### Treatment regimen and delivery

Patients received postoperative chemoradiotherapy with capecitabine and either IMRT (n = 133) or 3D-CRT (n = 51) (patients choice) following TME. The patients were irradiated in a prone position on a belly board, with bladder filling to reduce any intestinal toxicity. Simplified IMRT (sIMRT) is recommended in all patients in order to improve the dose coverage and reduce dose uncertainties during irradiation [27]. The treatment and delivery procedures for sIMRT were similar to those used for IMRT. However, sIMRT involved a five-radiation field, ≤5 segments per beam, a segment area ≥10 cm^2^ and ≥10 machine monitor units (MU) per segment. The delineation of the clinical target volume (CTV) was based on Roels’s guidelines and included the tumor bed and pelvic lymphatic area, such as the sacrum, the presacral space, the posterior walls of the bladder and prostate or vagina, and the internal iliac lymph nodes without inguinal lymph nodes [28].

The treatment field has been described previously in the phase I study [21]. Briefly, a 7-mm left-right (LR), 10-mm anteroposterior (AP) and 10-mm superior-inferior (SI) expansion of the CTV was used to create the planning target volume (PTV). A total irradiation dose of 50 Gy without simultaneous boost to the 95% PTV was delivered in 2-Gy daily fractions, from Monday to Friday, over a 5-week period. Organs at risk (OARs) were contoured, including the intestine, bladder and uterus; however, the irradiation dose was allowed to exceed 50 Gy in limited volumes of the small bowel and colon, if they were adjacent to the PTV, but it was not allowed to exceed 52 Gy. The bladder V50 dose limit was below 50%.

All treatment plans were generated using an inverse IMRT planning system developed by the PHILIPS Corporation, either the Pinnacle^3^ Version 7.4 or Version 8.0 planning systems. Treatment was delivered using a step-and-shoot technique with 6-MV photons. Portal image or cone-beam CT was acquired daily during the first week, and thereafter once a week.

Chemotherapy consisted of two cycles of oral capecitabine, which was 1600 mg/m^2^/d, administered twice daily from day 1–14 of the radiotherapy, followed by a 7-day rest. The second cycle was continued for the remaining radiotherapy. The first dose of capecitabine was given approximately 2 h before radiotherapy; the second dose was given approximately 12 h between radiotherapy treatments. After the chemoradiotherapy had been completed, 4 to 6 cycles of oxaliplatin-based adjuvant chemotherapy was recommended to patients with high-risk stage II or III disease, referring to patients with positive nodes, tumor nodules, poorly differentiated, or T4 disease. In total, 119/184 (64.7%) patients received adjuvant chemotherapy. The majority of these (108, 90.8%) were treated with oxaliplatin-based chemotherapy; the remaining 11 patients (9.2%) received capecitabine chemotherapy alone. The median number of cycles was six.

### Patient follow-up

Patients were evaluated weekly during treatment, 1 month after the completion of treatment, every 3–6 months for the first 3–5 years and annually thereafter. The median follow-up was 47 months (range, 3 to 91 months). Acute toxicity was scored according to the Common Terminology Criteria for Adverse Events (CTCAE v. 3.0), and late toxicity was graded according to the Late Effects in Normal Tissue—Subjective, Objective, Management and Analytic (LENT-SOMA) system.

OS was measured from the date of surgery to the date of death from any cause, or to the last follow-up date. DFS was measured from the date of surgery to any type of recurrence, or to the date of death as a result of rectal cancer. LRC was measured from the date of surgery to locoregional recurrence or the last follow-up.

### Statistical analysis

Patient characteristics between subgroups were compared using the chi-square test. Survival rates were calculated using the Kaplan-Meier method to construct survival curves. The log-rank test was used to compare survival outcomes. Cox regression was used for multivariate analysis of independent prognostic factors for survival.

## Results

### Patient characteristics

The clinical features of patients are presented in Table 1. The ratio of males to females was 1.3:1. The median age was 57 years (range, 27–75 years). According to the American Joint Committee on Cancer (AJCC, 6th edition) staging system, 90 (48.9%) patients had stage II disease and 94 (51.1%) patients had stage III disease. Pathological T4 diseases were reported in 2 (1%) patients. A total of 114 (62.0%) patients presented with mid- or low-tumor location. Poor tumor differentiation was reported in 25 (13.6%) patients, and lymphovascular invasion and tumor nodules were reported in 8 (4.3%) and 30 (16.3%) patients, respectively. The median time interval between surgery and radiotherapy was 1.4 months (0.5 to 3 months).

**Table 1:** Clinical characteristics of the study patients with stage II/III rectal cancer.

Characteristics		N (%)
Sex		
Male		104 (56.5)
Age (years)		
Median	57	
Range	27–75	
ECOG score		
0–1		172 (93.5)
2		12 (6.5)
Tumor level		
≤5 cm		70 (38.0)
5–10 cm		69 (37.5)
>10 cm		45 (24.5)
T stage		
T1–T2		19 (10.3)
T3–T4		165 (89.7)
N stage		
N0		90 (48.9)
N1		60 (32.6)
N2		34 (18.5)
Stage (AJCC)		
II		90 (48.9)
III		94 (51.1)
Operation type		
APR		42 (22.8)
LAR		142 (77.2)

**Abbreviations:** ECOG, Eastern Cooperative Oncology Group; AJCC, American Joint Committee on Cancer, 6^th^ edition; APR, abdominoperineal resection; LAR, low anterior resection.

### Outcome and prognostic factors

**Fig 1:**
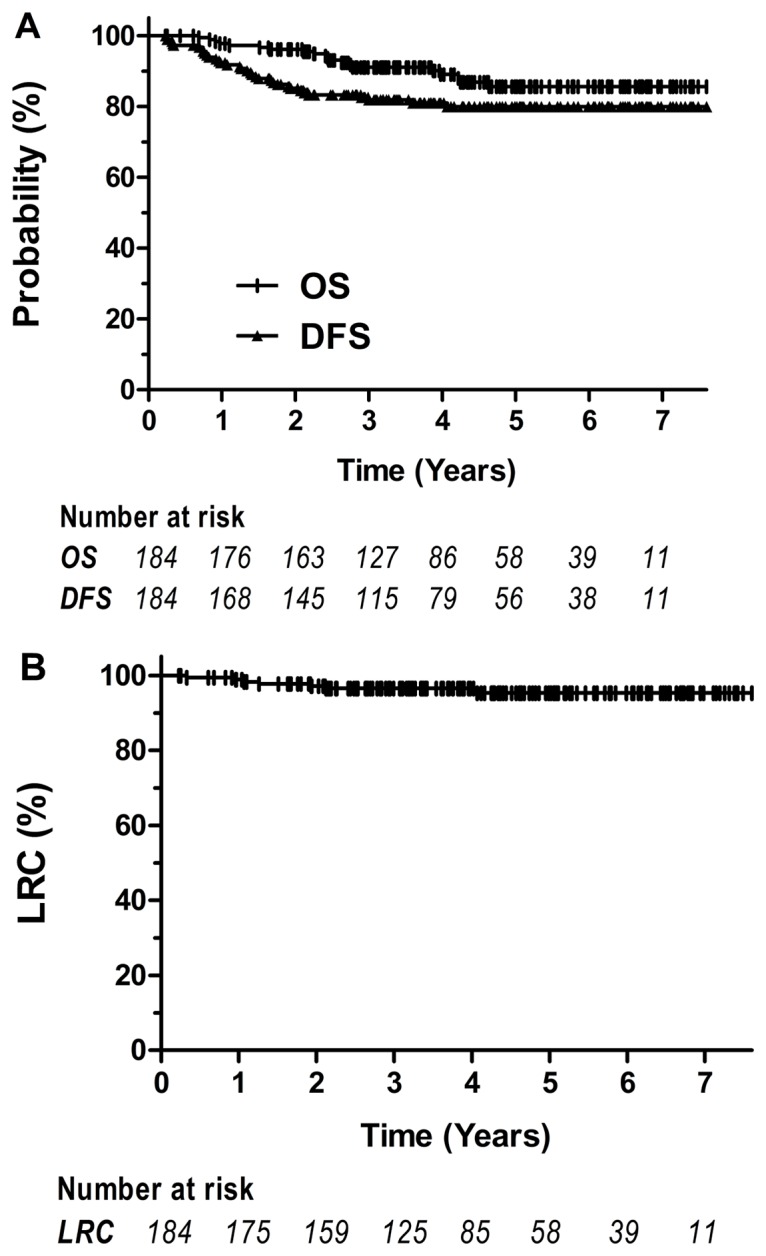
Overall survival (OS), disease-free survival (DFS) and locoregional control (LRC) rates for all patients undergoing postoperative chemoradiotherapy with capecitabine and concurrent intensity-modulated radiotherapy (IMRT)/three-dimensional conformal radiotherapy (3D-CRT).

Based on the median follow-up time of 47 months, with a range from 7 to 77 months, the 5-year OS, DFS and LRC rates were 85.1%, 80% and 95.4%, respectively (Fig 1). At the time of last follow-up, 21 (11.4%) patients had died of rectal cancer, and 7 (3.8%) patients were still alive with disease. Twelve patients (6.5%) were lost and included in the analysis.

Clinical features were evaluated to determine their prognostic significance on OS, DFS and LRC rates (Table 2). Univariate analysis revealed that patients with poor or moderately poor differentiation, advanced stage II or III disease, N2 stage disease or tumor nodules had poorer OS. Advanced stage disease and N2 stage disease were also significant prognostic factors for DFS. However, none of the clinicopathologic features were significant prognostic factors for LRC rate, possibly due to the small number of events. Cox regression analysis indicated that advanced N stage was the only poor prognostic factor for OS and DFS rates (Table 3).

**Table 2:** Univariate analysis showing the prognostic significance of clinicopathologic features on the outcomes of the 184 patients recruited in this study.

Variables	5-year OS		5-year LRC		5-year DFS	
	%	*P*	%	*P*	%	*P*
Age		0.582		0.293		0.99
≤60	86.2		97.2		80.3	
>60	82.8		92.1		79.1	
Stage (AJCC)		0.021		0.725		0.035
	91.4		96.5		86.7	
	79.4		94.6		73.6	
N stage		0.002		0.125		<0.001
N0	91.4		96.5		86.7	
N1	85.2		97.2		83.3	
N2	69.2		89.9		56.0	
T stage		0.712		0.807		0.636
T1–T2	77.6		91.7		80.5	
T3–T4	86.6		96.7		80.1	
Tumor grade		0.05		0.958		0.079
Well	83.1		94.7		78.2	
Moderately	87.9		96.3		83.3	
Poorly	71.1		91.7		64.9	
Tumor nodules		0.015		0.295		0.056
No	87.8		95.9		82.1	
Yes	70.1		93.0		68.8	

**Abbreviations:** OS, overall survival; LRC, locoregional control; DFS, disease-free survival; AJCC, American Joint Committee on Cancer, 6^th^ edition

**Table 3:** Multivariate analysis showing the prognostic significance of clinicopathologic features on the outcomes of the 184 patients recruited in this study.

Variables	OS		LRC		DFS	
	HR(95%CI)	*P*	HR(95%CI)	*P*	HR(95%CI)	*P*
Age	0.78(0.29–2.05)	0.608	2.03(0.39–10.59)	0.403	0.65(0.31–1.38)	0.261
Gender	0.51(0.19–1.33)	0.167	3.13(0.54–18.14)	0.202	0.69(0.34–1.4)	0.301
Operation	0.91(0.16–5.3)	0.919	0.61(0.02–20.79)	0.786	0.67(0.19–2.37)	0.532
T stage	0.88(0.22–3.48)	0.852	0.75(0.06–9.49)	0.823	1.67(0.47–5.91)	0.430
N stage	3.21(1.09–9.48)	0.035	6.08(0.6–61.59)	0.128	4.26(1.76–10.34)	0.001
Differentiation	1.86(0.69–5.05)	0.223	0.85(0.16–4.58)	0.851	1.7(0.8–3.62)	0.170
Stage	0.5(0.07–3.66)	0.491	0.05(0.00–3.90)	0.175	0.27(0.05–1.35)	0.111
Tumor level	0.64(0.26–1.61)	0.346	0.71(0.12–4.06)	0.700	0.68(0.35–1.31)	0.251
Lymphovascular invasion	1.91(0.39–9.35)	0.427	0.000	0.990	1.46(0.42–5.09)	0.552
Tumor nodules	1.9(0.59–6.11)	0.284	2.69(0.35–20.83)	0.342	1.46(0.62–3.43)	0.382

**Abbreviations:** RT, radiotherapy; OS, overall survival; LRC, locoregional control; DFS, disease-free survival; HR, hazard ratio; CI, confidence interval

### Tolerance and acute toxicity

None of the patients developed grade 5 acute toxicity (Table 4). Grade 3 and 4 toxicities were observed in 52 (28.3%) patients during chemoradiotherapy. The most common of these were diarrhea (42/184, 22.8%), leucopenia (7/184, 3.8%), tenesmus (5/184, 2.7%), dermatitis (4/184, 2.2%) and fatigue (1/184, 0.5%). Dose reduction, due to grade 3 and 4 toxicities was carried out in 19 (10.3%) patients who were receiving adjuvant chemotherapy. Radiotherapy or capecitabine were only interrupted or discontinued in 9 (4.9%) and 11 (6%) patients, respectively, due to treatment-related toxicity. Among them, most patients continued treatment after side effects alleviated. Only 2 patients with stage IIA received pelvic irradiation with dose less than 45 Gy (1 with 34 Gy and another with 44 Gy) and suffered distant metastases at 0.7 year and 1.6 years after surgery, respectively. For patients with treatment interruption, no loco-regional relapse was observed. Pelvic radiation doses ≥45 Gy were received by the majority of patients (182, 98.9%), indicating high tolerance of postoperative capecitabine with concurrent IMRT/3D-CRT.

**Table 4:** Incidence and type of acute toxicity developed in patients receiving postoperative chemoradiotherapy with capecitabine and concurrent IMRT/3D-CRT.

		Grade;	*N* (%)	
Toxicity	1	2	3	4
Fatigue	51 (22.4)	3 (1.6)	1 (0.5)	0 (0)
Skin reaction	57 (31)	28 (15.2)	4 (2.2)	0 (0)
Anorexia	54 (29.3)	5 (2.7)	0 (0)	0 (0)
Nausea	32 (17.4)	4 (2.2)	0 (0)	0 (0)
Vomiting	5 (2.7)	2 (1.1)	0 (0)	0 (0)
Diarrhea	33 (17.9)	35 (19)	41 (22.3)	1 (0.5)
Tenesmus	49 (26.6)	16 (8.7)	5 (2.7)	0 (0)
Body weight decline	18 (9.8)	2 (1.1)	0 (0)	0 (0)
Food-hand syndrome	0 (0)	1 (0.5)	0 (0)	0 (0)
Leucopenia	72 (39.1)	42 (22.8)	7 (3.8)	0 (0)
Thrombocytopenia	7 (3.8)	2 (1.1)	0 (0)	0 (0)
ALT elevation	10 (5.4)	0 (0)	0 (0)	0 (0)
TBIL elevation	8 (7.2)	2 (1.1)	0 (0)	0 (0)

**Abbreviations:** ALT, aminase; TBIL, total bilirubin level; IMRT, intensity-modulated radiotherapy; 3D-CRT, three-dimensional conformal radiotherapy.

### Late toxicities

Late toxicity was defined as that which occurred ≥3 months after radiotherapy. Only 9 (4.9%) patients presented with grade 3 or 4 late toxicities, including neurotoxicity (n = 1) and gastrointestinal toxicity (n = 8). Other severe late complications or second malignancies were not observed in any of the patients. Colostomies were performed on 6 (3.3%) of the patients with gastrointestinal toxicity.

## Discussion

The purpose of this retrospective study was to evaluate the toxicity profile and survival outcomes of postoperative chemoradiotherapy with capecitabine and concurrent IMRT/3D-CRT for patients with stage II or III rectal cancer. Our findings showed that TME followed by capecitabine with concurrent IMRT/3D-CRT resulted in excellent LRC (5 years, 95.4%) with favorable prognosis. Furthermore, the treatment was safe, well-tolerated and caused relatively few severe acute or late toxicities.

The 5-year incidence of locoregional recurrence in this postoperative chemoradiotherapy study (4.6%) was similar to those reported in preoperative chemoradiotherapy studies, including a German trial (6%) [8] and preoperative capecitabine-based trials (5%–6%) [10, 11, 29]. In contrast, the incidence was lower than most of those reported in other postoperative chemoradiotherapy studies (10%–15%) [8, 11, 30, 31]. The superior LRC rate in this study may be due to the improved quality assurance obtained with TME and better target coverage achieved with IMRT/3D-CRT. Although studies have demonstrated that TME can significantly reduce the incidence of locoregional recurrence [32–34], reports show that it remains high (20%–30%) in patients with stage III disease or distal tumors [33–36].

Recent studies have shown that IMRT provides superior target dose distribution and reduced normal tissue toxicity compared to conventional pelvic radiotherapy or 3D-CRT [22–25]; however, the use of IMRT in rectal cancer remains controversial in clinical practice. This may be due to the dose uncertainty induced by the prolonged treatment time and very small segments or fields. As such, sIMRT, which achieves a uniform dose distribution similar to that obtained with IMRT but with a shorter treatment time [27], has been recommended in our institution since 2005. Our data in this study has demonstrated that sIMRT/3D-CRT can achieve high LRC rates (>90%), which are similar or superior to those obtained with conventional radiotherapy, indicating that the sIMRT technique is safe for use in the clinic. Furthermore, the majority of patients in our study received full-dose pelvic radiotherapy with IMRT/3D-CRT, demonstrating that high LRC rates can also be achieved with concurrent, postoperative, capecitabine-based chemoradiotherapy. In addition, several recent randomized studies have found that compared to 5-FU, capecitabine-based concurrent chemoradiotherapy is better tolerated, associated with lesser toxicity and achieves similar OS rates and better DFS rates [10, 11, 29, 37].

The incidence of grade 3 or higher acute toxicity for preoperative chemoradiotherapy for rectal cancer ranges from 6% to 27% [8, 29, 37], increasing to 16%–40% for postoperative chemoradiotherapy [8, 9, 29]. The incidence of grade 3 and 4 acute toxicity (28.3%) in this study was lower than the rates reported for postoperative chemoradiotherapy in many studies, such as the German and NSABR-R03 trials [8, 9], but higher than the rates reported for preoperative chemoradiotherapy [25, 29, 37]. Despite moderate acute toxicities, <5% of patients presented severe late toxicities (grade 3 and 4). Further confirmation that postoperative chemoradiotherapy with capecitabine and concurrent IMRT was well tolerated was demonstrated by the observation that approximately 95% of our patients received the prescribed concurrent treatment regimen as planned.

However, there is some limitations of this study due to the retrospective analysis. Patient selection bias may be contributed to the better LRC and OS.

In conclusion, this study demonstrated that patients with stage II and III rectal cancer can be treated safely and efficiently by postoperative chemoradiotherapy with capecitabine and concurrent IMRT/3D-CRT. Although further studies will be required to validate the benefits of precise target dose coverage with IMRT in postoperative chemoradiotherapy, our data shows that superior LRC and improved survival rates can be achieved with capecitabine and concurrent IMRT/3D-CRT following TME.
